# What do bereaved relatives of cancer patients dying in hospital want to tell us? Analysis of free-text comments from the International Care of the Dying Evaluation (i-CODE) survey: a mixed methods approach

**DOI:** 10.1007/s00520-022-07490-9

**Published:** 2022-12-23

**Authors:** Christina Gerlach, Miriam Baus, Emilio Gianicolo, Oliver Bayer, Dagny Faksvåg Haugen, Martin Weber, Catriona R. Mayland, ERANet-LAC CODE Core scientific group, Katrin Sigurdardottir, Marit Irene Tuen Hansen, Karl Ove Hufthammer, Wojciech Leppert, Katarzyna Wolszczak, Eduardo Garcia Yanneo, Vilma Tripodoro, Gabriel Goldraij, Christina Gerlach, Lair Zambon, Juliana Nalin Passarini, Ivete Bredda Saad, John Ellershaw, Grace Ting

**Affiliations:** 1 Interdisciplinary Palliative Care Unit, Department of Medicine, University Medical Center of Johannes Gutenberg University, Mainz, Germany; 2 Department of Palliative Care, Heidelberg University Hospital, Heidelberg, Germany; 3 Institute of Medical Biostatistics, Epidemiology and Informatics (IMBEI), University Medical Center of Johannes Gutenberg University, Mainz, Germany; 4 Institute of Clinical Physiology, National Research Council, Lecce, Italy; 5 Regional Centre of Excellence for Palliative Care, Western Norway, Haukeland University Hospital, Bergen, Norway; 6 Department of Clinical Medicine K1, University of Bergen, Bergen, Norway; 7 Department of Oncology and Metabolism, University of Sheffield, Sheffield, UK; 8 Palliative Care Unit, University of Liverpool, Liverpool, UK

**Keywords:** (MeSH): End of life care, Palliative care, Qualitative research, Quality of healthcare, Gratitude, Supportive care, Narrative medicine

## Abstract

**Purpose:**

We conducted an international survey of bereaved relatives of cancer patients dying in hospitals in seven countries, with the aim to assess and improve the quality of care. The survey used the i-CODE (International Care of the Dying Evaluation) questionnaire. Here, we report findings from the free-text comments submitted with the questionnaires. We explored for topic areas which would potentially be important for improving the quality of care. Further, we examined who reported free-texts and in what way, to reduce bias without ignoring the function the free-texts may have for those contributing.

**Methods:**

We used a combined qualitative-quantitative approach: logistic regression analysis to study the effect of respondents’ socio-demographic characteristics on the probability of free-texts contributions and thematic analysis to understand the free-text meaning. The primary survey outcomes, (1) how frequently the dying person was treated with dignity and respect and (2) support for the relative, were related to free-text content.

**Results:**

In total, 914 questionnaires were submitted; 457/914 (50%) contained free-text comments. We found no socio-demographic differences between the respondents providing free-texts and those who did not. We discovered different types of free-texts (“feedback,” “narrative,” “self-revelation”) containing themes of which “continuity of care,” “the one person who can make a difference,” and “the importance of being a companion to the dying” represent care dimensions supplementing the questionnaire items. A free-text type of grateful feedback was associated with well perceived support for the relative.

**Conclusion:**

Bereaved relatives used the free-texts to report details related to i-CODE items and to dimensions otherwise not represented. They highlighted the importance of the perceived support from human interaction between staff and the dying patient and themselves; and that more than professional competence alone, personal, meaningful interactions have profound importance.

**Supplementary Information:**

The online version contains supplementary material available at 10.1007/s00520-022-07490-9.

## Introduction

Hospital remains a common place of death. Although many cancer patients wish to die at home, a substantial group will, for good reasons, need hospital care in their final days [1, 2]. Hence, enabling hospital staff to provide quality care and support for dying patients and their families continues to be critically important [3]. Bereaved relatives’ accounts remain the best approach to assess the quality of end-of-life care [4, 5]. The subjectivity of the experience captured in relatives’ self-reports must be critically reflected upon but is a strong representation of what remains in their memory of the experience of death.

Overall, memories that were emotionally relevant for the relatives are probably shared [6–8], which is why it can be assumed that the topics mentioned are particularly important for the participants.

Listening to the bereaved relatives’ voices is critically important both for exploring future service users’ preferences and for studying the overall impact of the care in the patients’ dying phase on their relatives [9, 10]. When a close relative is dying, people are confronted with their own mortality, which is a threat according to terror management theory [11]. This theory also suggests social attachment as a possible way of support [12]. Thus, identification of suitable elements to provide support for the relatives as well as promote dignity of the dying patient is equally important.

In addition to *what* is reported, *how* it is reported can also be informative. Relatives’ gratitude may reflect good care [13, 14] and can serve a dual purpose: to offer feedback that motivates the staff about the care they provide; and as a way to support coping in the midst of a crisis, i.e., to help relatives cope with their grief [15].

Following the 2nd call of the Network of the European Union and the Community of Latin American and Caribbean States on Joint Innovation and Research Activities (ERANet-LAC) addressing “Improving the quality of care and quality of life of dying cancer patients,” we conducted a mortality follow-back survey with partners from four European (Germany, Norway, Poland, UK) and three South-American (Argentina, Brazil, Uruguay) countries. The objectives of the ERANet-LAC CODE project were to systematically assess the current quality of care and support for dying cancer patients and their families [16–18], to identify areas requiring improvement, and to test and evaluate quality improvement measures. The primary outcomes of the i-CODE-survey were treating the dying patient with respect and dignity and support for the relatives. Here, we report findings from the free-text comments provided by bereaved relatives as part of the survey, with the aim to use the learning from these to improve care and support for dying patients and their relatives. We explored the free-text comments to further understand and supplement topic areas which could be important for improving the quality of care. We wanted to know not only *what* the relatives reported in the free-texts, but also *how* they did it, and what we can learn from these aspects. Further, we related these to the primary quantitative outcomes of the survey: respect and dignity for the patient and support for the relative.

## Material and methods

### Methodological orientation and theory

The main focus of the free-text analysis was the perceptions of care by relatives, present during the last days of life with a cancer patient in hospital. We explored the content and way in which bereaved relatives expressed their own and their family members’ needs beyond validated questionnaire items. Quantitative methods were used to frame the context and control reporting bias. Epistemologically, our approach is based on subtle realism, which defines reality as independently existing but accessible by observation of individual perceptions and interpretations in a defined context [19, 20]. Recognizing not only the content but also the narrative style of the free-texts belongs to the principles of narrative medicine [21]. A mixed methods approach was used to triangulate the qualitative content analysis with the demographic data and statistical survey outcomes [22].

### Study setting and respondents

Recruitment occurred within 22 hospitals in 7 countries as previously detailed; hence, a brief overview is provided [16]. Respondents were adults (≥ 18 years), able to provide informed consent, who were the relative to an adult cancer patient who died an expected death in one of the participating hospitals. “Relative” was defined as the next-of-kin recorded in the patient’s hospital record and was present at least some of the time during the dying phase.

### Data collection

The data collection tool of the study was the i-CODE, a 42-item self-completion questionnaire evaluating symptom control, nursing and medical care, emotional and spiritual support, information and decision-making, environment, preparation, and support at the actual time of death and immediate post-bereavement period [17, 18, 23]. The relatives received the questionnaire 6–8 weeks post bereavement. All free-text comments in the provided space at the end of the questionnaire, and texts submitted as an attachment to the questionnaire, were included for analysis. The translations contain unintentionally differing prompts for free-text contribution (Supplement 1).

### Qualitative analysis

**Fig. 1:**
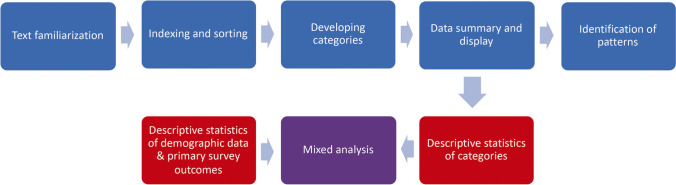
Methodological process of the qualitative content analysis (blue) and quantitative analysis (red). Stepwise approach of the framework analysis combined with statistical analysis. Primary survey outcomes were respect and dignity for the dying and support for the relative

Framework analysis is based on a matrix, which inductively organizes the data and identifies themes within and across individual free-texts by the use of five related steps [24–26]. The analytic process was repeated and re-repeated until no new information was obtained (Fig. 1). The study’s core international research team contributed to semantic clarification, and all categories were consented across the 7 countries, embracing code names, definition of each category, and the choice of individual national anchor quotations. Two researchers independently coded the free-texts (C.G., M.B.). Inconsistencies between coders were reconsidered and alternative interpretations incorporated into the analysis. All quotes from the same category were displayed in a framework, and written summaries of the quotes allowed to discover patterns between individual free-texts. Interim results of the analytic process were further tested during discussions with colleagues. Free-text about topics not related to the aim of the study were considered with special attention to avoid omission of important observations. The free-texts were managed in MS Word and MS Excel spreadsheets, and advanced analysis was assisted by qualitative data analysis software (VERBI Software, MAXQDA Analytics, Version 11).

### Statistical analysis

We compared relatives’ demographic data (country, age, gender, religious affiliation, relationship to the deceased, place of care) and primary survey outcomes (respect and dignity for the dying patient; support for the relative) between those who provided free-texts and those who did not. Differences were tested with the Chi-square-test with a two-sided significance level of 0.05. Logistic regression analysis was used to investigate the association between gender, age group, whether the patient died in a palliative care unit or other, and degree of relationship of the respondents, and the probability of respondents in providing a free-text. Odds ratios (OR) and associated 95% confidence intervals (95% CI) were computed. Statistical analysis was conducted with SAS Version 9.4 (SAS Institute, Inc., Cary, NC, USA).

### Mixed methods

Free-text categories from the qualitative analysis that were considered to be particularly relevant for quality improvement were selected. To avoid multiple testing, the feedback categories “complaint”, “burden of negative experiences”, and “suggestions and demands for improvement” were combined as “criticism of care” for the mixed-methods analysis, because all three categories have a negative connotation and provide a focus for improvement. “Criticism of care” was contrasted with the positive feedback categories “praise” and “gratitude”. We determined the frequencies of the categories and tested for correlation with the primary survey outcomes, i.e., “How much of the time was s/he treated with respect and dignity in the last two days of life by nurses/ by doctors?” and “Overall, in your opinion, were you adequately supported during his/her last two days of life?”, and demographic data, to identify response patterns contributing to a deeper understanding of the relatives’ perception of good end-of-life care for cancer patients in hospitals (Fig. 1) [22, 27]. The research team for the mixed-methods analysis consisted of a clinical scientist (CG) and an epidemiologist (OB) experienced in qualitative analysis, a statistician (EG), and a research assistant and undergraduate medical student (MB).

## Results

### Sample characteristics

A total of 914 completed i-CODE questionnaires were received. Free-text comments were included in 457/ 914 (50%) of the questionnaires (Table 1). Respondents providing free-text comments were mostly female (67.8%), aged under 60 years (56.3%) or between 60 and 80 years (38.5%), and spouse/partner (44.2%) or children (-in-law) (39.0%) of the deceased. The majority reported they were “Christian” (78.3%). Logistic regression analysis showed no difference between respondents providing free-text comments and those who did not, in terms of age (OR: 0.96; 95% CI 0.70–1.34), gender (OR: 1.09; 95% CI 0.83–1.45), and relationship (OR: 1.20; 95% CI 0.85–1.70). Type and content of the free-texts were clearly triggered by the free-text prompts, which differed between countries (Supplement 1). We found no correlation between the demographic data and the positive and negative feedback categories (“gratitude” Supplement 2, and “praise” and “criticism of care” Supplement 3).

**Table 1:** Participant characteristics. Overview of the participants’ demographic data divided into the two groups free text yes/ no and the statistical significance

	Free text		*p* value
	Yes	No	
Total	457 (100.0%)	457 (100.0%)	
Country			< 0.0001
Germany Norway UK Brazil Argentina Poland Uruguay	116 (25.4%) 104 (22.8%) 77 (16.9%) 61 (13.4%) 42 (9.2%) 33 (7.2%) 24 (5.3%)	67 (14.7%) 90 (19.7%) 25 (5.5%) 44 (9.6%) 63 (13.8%) 67 (14.7%) 101 (22.1%)	
Gender (relative)			0.85
Male Female Missing data	140 (30.6%) 310 (67.8%) 7 (1.5%)	148 (32.4%) 302 (66.1%) 7 (1.5%)	
Age (relative)			0.81
< 60 60–79 ≥ 80 Missing data	257 (56.3%) 176 (38.5%) 20 (4.4%) 4 (0.9%)	253 (55.4%) 182 (39.8%) 16 (3.5%) 6 (1.3%)	
Religion (next-of-kin)			0.08
Christian Other religions No religion Missing data	358 (78.3%) 19 (4.2%) 74 (16.2%) 6 (1.3%)	345 (75.5%) 37 (8.1%) 67 (14.7%) 8 (1.8%)	
Relation to patient			0.71
Spouse/partner Children(-in-law) Other relations Missing data	202 (44.2%) 178 (39.0%) 74 (16.2%) 3 (0.7%)	209 (45.7%) 162 (35.5%) 83 (18.2%) 3 (0.7%)	
Palliative care unit			0.44
Yes No	106 (23.2%) 351 (76.8%)	116 (25.4%) 341 (74.6%)	

### Qualitative categories

The analytic process revealed a two-level system of categories. The first considered the type of free-text (Table 2): (I) feedback including gratitude, (II) narrative, and (III) self-revelation. The second identified 5 content categories with 13 subcategories, each of which had positive and negative manifestations (Table 3): (1) performance of care, (2) the human factor, (3) communication, (4) environment, and (5) the importance of being a companion to the dying.

**Table 2:** Typology hierarchy. Hierarchical portrayal of the typological categories and subcategories including the definitions of the categories

Category	Subcategory	Definition
Feedback	Praise	The text is used to highlight positive experiences made in the hospital or regarding the dying phase
	Suggestions/demands for improvement*	Ideas by the relative how the hospital/staff could improve its work. This could be a direct suggestion or provided in an indirect way, i.e., complaint about issues that enable conclusions to be made about how to improve patient care and support for the relatives
	Burden due to negative experience*	The relative feels an emotional burden caused by the experience of poor care or inappropriate circumstances (e. g., the organizational structures of the hospital) during the hospital stay. The poor care or inappropriate circumstances are explicitly reported as a distressing experience for the relative
	Gratitude	The relative uses the opportunity to explicitly say “thank you” or to express their gratitude for the care, to the team or to a certain person
	Complaint*	The text is used to report concrete negative experiences made in the hospital or regarding the dying phase, but the content of the free text does not allow for further interpretation regarding emotional processing, or improvement ideas
	Feedback to questionnaire	The relative suggests how the questionnaire could be improved
Self-revelation	Social context	The relative reveals personal information about himself/herself or the patient
	Coping with bereavement	The relative explains how s/he is dealing with the loss or how the time since the death has been for him/her
	Impact on health/psyche	The burden caused by being an informal caregiver has a negative impact on the relative’s health or emotional well-being. This impact can be described as (mental/ physical) exhaustion or sadness
	Rage over negative experience	The relative expresses strong feelings such as rage attributed to negative experiences about care, communication or hospital procedures
	“Kitchen table” wisdom	This category collects wisdom from real life experiences which the relative shares in the free-texts
Narrative	Chronicle	Chronological description or report of the patient’s history including hospital stay, therapies and decline
	Witnessing the imminently dying	Report of the imminently dying phase

*Summarized for the mixed-methods analysis to “criticism of care”; anchor quotations are provided in Suppl. 4

**Table 3:** Content hierarchy. Hierarchical portrayal of the content categories and subcategories including anchor examples to demonstrate the meaning of the category

Category	Subcategory	Anchor quotation
Performance of care	Competence	( +) “In general, I would like to say that even during the previous stay of my wife in [name of the hospital], the care provided by the doctors and all the nursing staff was very good, and I am sure that from a medical and nursing point of view everything, really everything, was done to achieve a recovery from the illness.” (Germany, Palliative Care Unit) ( −) “My husband was admitted to hospital because he needed pain relief. He had a lot of pain, and staff did not manage to get him pain free. Young, insecure and inexperienced nurses.” (Norway, Surgical Ward)
	Organization and administration of care, i.e., continuity of care, time, staffing	( +) “In the ICU the allocation of staff is far better.” (Germany, ICU) ( −) “The waiting times quickly listed: CT scan appointment for 10 a.m.—> 3.5 h of waiting, pick up by the transport company—> 3 h, finally arrived on the ward after 8 h.” (Germany, Oncology)
	Committed care	( +) “Just thank you to all the doctors, nurses and everyone else who contributed to my mother’s dignified death and who made the impression of fully devoting themselves to their job.” (Argentina, Oncology) ( −) “The patient gets his meal, and it is taken away again without anyone noticing that the patient has eaten near to nothing.” (Germany, ICU)
	Relative responsible for hospital care	( +) “I cared for my husband all the time while he was on the ward. I was there from dusk till long after dawn, I cared for him, washed him, fed him, gave him pills and went to the toilet with him.” (Germany, Oncology) ( −) “We as relatives had to point out an unnecessary CT scan of the seriously ill patient after a transfer (at weekend) from the [name of ward 1] to the [name of ward 2]. There had been a scan shortly before that and apart from additional stress for the coma patient there would have been no new findings. We had to watch that he got his drugs in time as well as paying attention to the expiration time of the transfusion (uncooled not longer than three hours).” (Germany, ICU)
The human factor	Support	( +) “In general, we received enormously good support and care as relatives in this situation, which made both of us safe and gave us good confidence in both doctors and, not least, nurses.” (Norway, Medical Ward) ( −) “My daughter and I sat the whole day at our husband’s/father’s bed before he died in the afternoon. We were not asked if we would like to have a little food or drink. We left the room and went downstairs to buy food and drink, but in such circumstances, one does not wish to leave one’s dear one. I therefore think it would have been nice if we had been asked if we wanted something to eat or drink.” (Norway, Medical Ward)
	Behavior	( +) “The last days the patient, as well as we as his relatives, were treated well and with respect, especially by the nurses.” (Norway, ICU) ( −) “We felt that a couple of the nurses /assistant nurses were not fit to work on such a ward. They were sharp and arrogant, we felt we bothered them.” (Norway, Medical Ward)
	The one person who can make a difference	( +) “I remember a blonde angel who listened to me a lot while she put an oxygen mask on my husband. He breathed better and she talked to me.” (Brazil, Medical Ward) ( −) “All of the doctors were amazing except for one who turned out to be the most obnoxious and insensitive person I have ever encountered in the medical profession. He made it quite clear that my uncle was on the wrong ward and basically, he could not do anything for him, I was under no illusion that my uncle was dying. The consultant showed no sympathy whatsoever and kept referring to my uncle as my dad (did not listen!). He should stick to his graphs and charts and statistics and stop speaking to patients, families and nurses in his vile manner.” (UK, Surgical Ward)
	Acknowledgement of patient’s wishes	( +) “The palliative care team accompanied me during the entire process and fulfilled everything we wished for.” (Uruguay, Emergency Room) ( −) “My mother was taken to x-ray again because they suspected pneumonia. Again, strong pain, screaming, suffering. And that even though my mother said explicitly that she wanted to be treated palliatively only.” (Germany, Palliative Care Unit)
Communication	Information	( +) “My father felt to be in good hands, because he always received a competent, comprehensive, and first of all honest answer to his questions. The counseling by the mentioned colleagues considering tests and current as well as planned therapies was comprehensive and empathic and adequate to the situation.” (Germany, Oncology) ( −) “It can never be too much information when one’s closest suddenly gets seriously ill, I missed more information about the disease and especially the anxiousness that was unbearable the last four weeks on the palliative care unit both for the patient and the next-of-kin.” (Norway, Palliative Care Unit)
	Involvement in decision-making	( +) “I applaud the caring staff for including me in the discussion about her care while she was in the intensive care unit (gave me the opportunity to be included).”(Germany, ICU) ( −) Through a long disease trajectory my opinion is that involvement of relatives has been far too poor.” (Norway, Medical Ward)
Environment	Ambience	( +) “Particularly positive about the palliative ward was: the overall atmosphere, for the relatives as well, with garden, living room, etc., which could be used at any time. […] That the patients could go into the garden with their beds. That, despite everything, it is a lively ward where it is okay to bake cakes and laugh.” (Germany, Palliative Care Unit) ( −) “The rooms are too small. More space is needed. There was no ‘goodbye room’. After his death, the patient was lying in the same room, and behind the partition lay another conscious patient.” (Poland, Palliative Care Unit)
	Place of death	( +) “He wanted to die at home and was home as long as he/we controlled his pain. But when that was impossible, the hospital was the only relevant place for him. […] In this case the hospital did all they could to give him a dignified death, e. g. with pain relief. That was what he wanted.” (Norway, Surgical Ward) ( −) “My husband should have been at a palliative care unit having an expertise that would have given my husband a better ending to his life and cared for us as relatives.” (Norway, Surgical Ward)
	Privacy	( +) “I am especially grateful to the [name of the hospital] that my husband could stay in a single-bed room on his ward and was not transferred. And that we could say good-bye there.” (Germany, Oncology) ( −) “You can never have perfection, but a single room would have been appreciated and we are aware of "availability" not always possible, but it doesn’t detract from the care and love received.” (UK, Palliative Care Unit)
Importance of being a companion to the dying		( +) “We want to highlight very positively that we (being her 3 children) were allowed to stay with her in the last 6 days of her life, when it was already foreseeable that she would die. Thus, we were all together day and night and got the opportunity to have quality time, and to bid farewell.” (Germany, Medical Ward) ( −) “However, it is a great burden to me that I was not informed in time of the death of my [wife] during her last stay in the [name of the hospital] so that I could not support her during the last hours of her life.” (Germany, Haematology Ward)

(+) positive manifestation; (−) negative manifestation of the category; *ICU*, intensive care unit.

Inter-coder reliability correlation kappa was substantial (kappa = 0.71) [28].

**Fig. 2:**
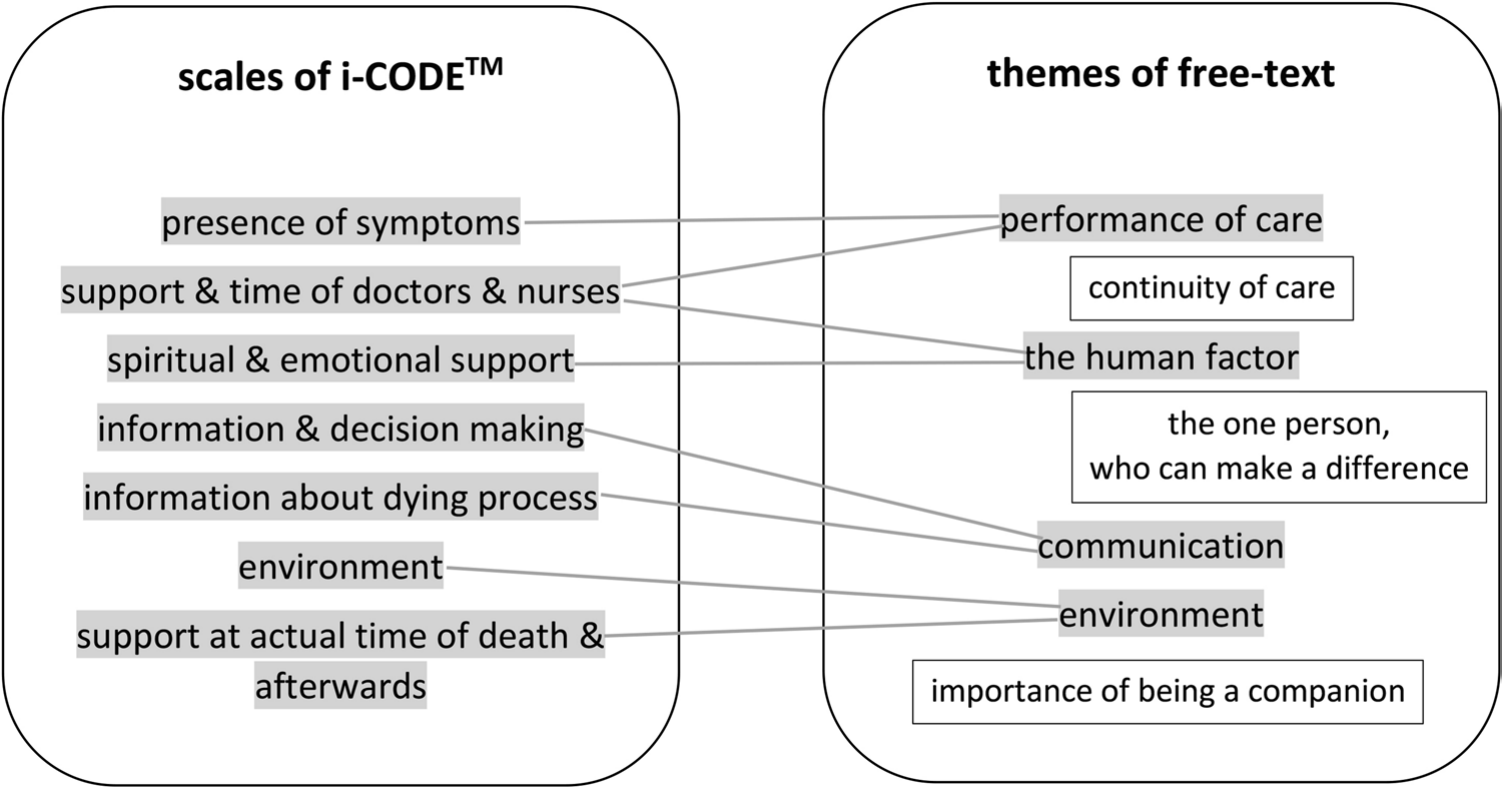
Additional dimensions. Comparison of the CODE themes (left) with the free-text themes (right) with framed themes, which were not covered by the questionnaire items

Most of the themes that the relatives addressed within the free-texts were congruent with the i-CODE-topics, but provided more detail. As complementing themes to the existing i-CODE-topics, we found (Fig. 2):

Continuity of care.

The one person who can make a difference.

The importance of being a companion to the dying person.

Although “continuity of care” often referred to the time before the dying phase, the relatives found it important to address as it could subsequently impact on the quality of care of the dying.

Continuity meant knowing the doctors and being treated by someone or a team who knew the patient’s history and needs. Of note, the relatives perceived lack of continuity as uncoordinated care.

“Continuity is important regarding who should provide information, during the diagnostic as well as the treatment periods. At the start of the trajectory there were too many different doctors.” (Norway, Medical Ward)

“The one person who can make a difference” and “importance of being a companion to the dying person” will be discussed with the relatives’ perceptions about being supported, which evoked their gratitude.

### Gratitude as report type

The coding-process revealed that relatives often used the free-text space almost as a way to provide a letter of appreciation, therefore gratitude evolved as a typological category. It was defined as the explicit expression of gratefulness by the relative, either toward one person in particular or toward the entire care team. In contrast to thank-you letters, this type of free-text contained specific reflective accounts of gratitude rather than polite general statements.

**Fig. 3:**
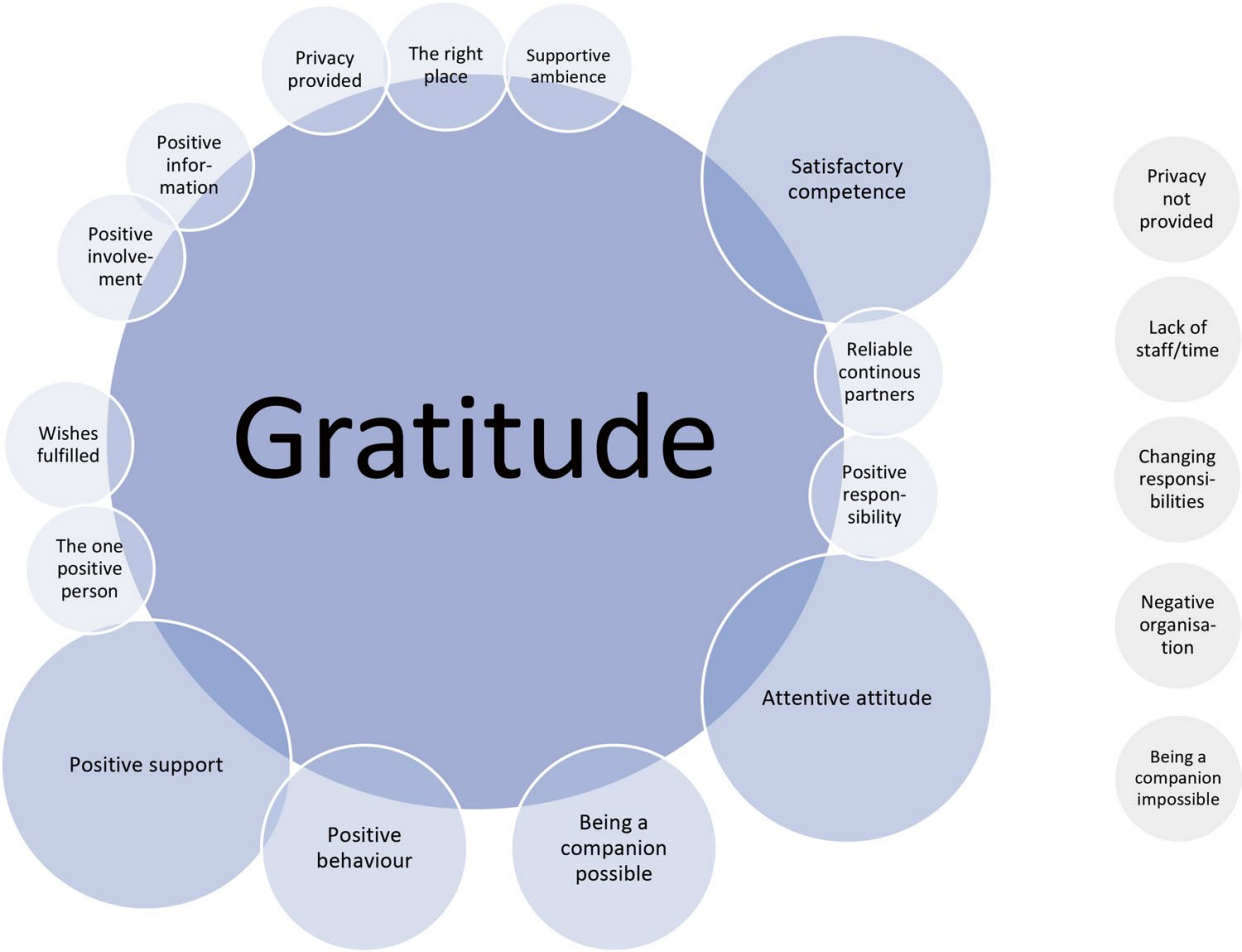
Themes of gratitude. Chart of the overlapping of content categories with the typological category gratitude. The size of the bubbles correlates in a simplified manner with the number of overlaps: small < 10 overlaps, medium 10–20 overlaps, big > 20 overlaps; negatively connoted categories with overlaps are displayed on the right

As gratitude may be a psychological concept which supports resilience and helps to cope with loss, we decided to identify the changeable factors of care in the dying phase that are reported in the context of gratitude. These factors of care were defined by those content categories named in the gratitude type of free-texts. Most frequently stated content aspects were (I) competent care (positive manifestation of the “competence” category), combined with (II) an attentive attitude (positive manifestation of the ‘committed care” subcategory) and (III) good behavior of staff (positive manifestation of the subcategory within “the human factor”), (IV) the importance for relatives of being a companion to the dying person, and (V) support for the relative (positive manifestation of the subcategory within “the human factor”) (Fig. 3).

(I)

“They took very, very good care of my mother and her relatives. Her strong pain and her suffering were improved for the most part and she was able to die in dignity. Again, many thanks to the whole team of the palliative care ward.” (Germany, Palliative Care Unit)

(II)

“Just thank you to all the doctors, nurses and everyone else who contributed to my mother’s dignified death and who made the impression of fully devoting themselves to their job.” (Argentina, Oncology Unit)

(III)

“Even though the intervention or support was not necessary, the entire staff was very respectful; even when they entered the room while we were praying or singing in order to prepare the way and create a peaceful atmosphere, the staff was attentive and respectful, never interrupted us; they did not even make us leave the room when the room was cleaned, but cleaned quickly, but still properly, around us.” (Argentina, Oncology Unit)

(IV)

“… it was arranged that we could be staying with him the whole time (6 days and nights).…Eternally grateful.” (Norway, Oncology Unit)

(V)

“As sad as the last hours were after all I am grateful to the doctors and nursing staff for making it possible for us and supporting us in accompanying my mother.” (Germany, ICU)

Negatively connoted content was also mentioned in the context of gratitude, but the relatives were grateful even though some negative aspects were experienced. Their motivation to combine complaints and gratitude may have been to encourage care improvements by concrete naming of care deficiencies (Fig. 3).

“Often they seemed to work in difficult circumstances and with limited resources. Thank you!” (UK, Medical Ward)

There was no correlation between a gratitude type of free-text and care provided in a palliative care unit, but a clear correlation with the questionnaire item asking about the relative’s perception of being supported (Q31) (*p* < 0.001; Supplement 2).

### Support experiences inform gratitude

Considering the correlation between gratitude and feeling supported (Supplement 2) and because “support for the relative” was not only a questionnaire item but also a theme of the free-texts (Table 3), we further explored the elements which contributed to this support. Those respondents who felt unsupported clarified details of their negative experiences by writing a free text in most cases (63/96; 65.6%). Overall, 788/914 (86.2%) of the responding relatives felt supported (Table 4). Thus, the free-texts could be used to describe the interventions which were perceived as supportive.

**Table 4:** Primary outcome of the i-CODE and association with free-text contributions. Overview of the overall outcome assessed with the i-CODE divided into the two groups free text yes/ no and the statistical significance

	Free-text		*p* value
	Yes	No	
Respect/ dignity (nurses)^1^			0.0004
Yes No Missing data/I do not know	413 (90.4%) 40 (8.8%) 4 (0.9%)	436 (95.4%) 13 (2.9%) 8 (1.8%)	
Respect/dignity (doctors)^2^			< 0.0001
Yes No Missing data/I do not know	387 (84.7%) 46 (10.1%) 24 (5.3%)	433 (94.8%) 10 (2.2%) 14 (3.1%)	
Support for the relatives^3^			0.0001
Yes No Missing data/I do not know	372 (81.4%) 63 (13.8%) 22 (4.8%)	416 (91.0%) 33 (7.2%) 8 (1.8%)	

^1^Q30: How much of the time was s/he treated with respect and dignity in the last two days of life? (nurses)

^2^Q30: How much of the time was s/he treated with respect and dignity in the last two days of life? (doctors); “Yes” for “always” and “most of the time”; “No” for “some of the time” and “never”

^3^Q31: Overall, in your opinion, were you adequately supported during his/her last two days of life?

The thematic complex of support was defined as active and intentional help by staff members that made the relative feel encouraged and comforted.

Communication was fundamental for understanding and enabled the relative to make informed decisions. Sensitive information about the dying phase provided comfort to the relative and seemed to be a prerequisite for being a companion to the dying relative.

“We were aware of the patient's medical condition at all times. In the intensive care unit as well as in the normal ward of the [name]. The doctors informed us frankly while staying friendly and considerate.” (Germany, ICU)

“We want to highlight very positively that we (being her 3 children) were allowed to stay with her in the last 6 days of her life, when it was already foreseeable that she would die. Thus, we were all together day and night and got the opportunity to have quality time, and to bid farewell.” (Germany, Medical Ward)

In contrast, delays and interruptions in the flow of information affected the relatives' trust in the staff, especially when inadequately prepared for the dying phase or death.

“One point of criticism regarding the care in the last days of life is that between being notified by the doctor that my mother was at her final stage of life and her death, less than 24 hours went by. The same doctor did not tell us that it would be a matter of hours, which would have been very valuable for the family.” (Argentina, Oncology)

Several relatives reported the missed opportunity to be at the bedside prior to death underlining the subsequent importance of this in their grief.

“However, it is a great burden to me that I was not informed in time of the death of my wife during her last stay in the [name of the hospital] so that I could not support her during the last hours of her life.” (Germany, Haemato-oncology Unit)

Knowing that the patient received good care and being confident in the available support represented further positive facets of the category “support”: certain that they were not alone created a feeling of safety.

“At this point the crossover of nurses were coming and all the lovely nurses who had been there looking after mom so well. They offered us support and love and didn’t leave our side until we left the hospital.” (UK, Medical Unit)

It was all the worse when the expectations of the relatives did not match with the reality of care.

“Before giving strong sedating medication to the patient for the first time intravenously or subcutaneously relatives should be informed about possible reactions of the patient. Otherwise his condition could frighten them. It is intimidating if the patient wakes up from sedation and experiences shortness of breath, is extremely congested and cannot expectorate.” (Germany, Palliative Care Unit)

Acknowledging the presence of the relative and his/her needs and assuring privacy were also ways of supporting the relative. Small gestures had a big impact.

“They offered us a single room and (…) the entire family could be present without a maximum of visitors which allowed that my husband’s siblings could come from other provinces in order to say goodbye and also our children could be there. For the night we got permission that two persons could stay with him and they also paid attention to my needs the moment he died.” (Argentina, Oncology)

By way of contrast, where this was not recognized, feedback reflected the impact on the relatives.

“They were sharp and arrogant, we felt we bothered them.” (Norway, Medical Ward).

In some cases, relatives reported about one particular person who provided support, highlighting the individual who had made a difference.

“Dr [Name] was the one who gave me the news of my uncle´s death. She stood beside me and gave me support until my husband arrived.” (Brazil, ICU)

In some cases, the behavior of this one person even served to overshadow experiences of deficit care.

“Eventually, I raved. Finally, a sister came (thank you) with a morphine injection, after hours!!! She cared for my mother very lovingly and took care of her, because she was already lying in the hallway since 7 p.m.” (Germany, Palliative Care Unit)

The reasons why one person was experienced as supportive seemed to be that this person combined all the supportive qualities in him-/herself. On occasion, however, one specific individual could leave very negative, emotional memories.

“All of the doctors were amazing except for one who turned out to be the most obnoxious and insensitive person I have ever encountered in the medical profession. He made it quite clear that my uncle was on the wrong ward and basically he could not do anything for him, I was under no illusion that my uncle was dying. The consultant showed no sympathy whatsoever and kept referring to my uncle as my dad (did not listen!).” (UK, Surgical Ward)

### Association between free-text types and main results of the i-CODE questionnaire

We triangulated the results from the primary outcome items of the questionnaire (respect and dignity of the dying, and support for the relative) with the free-text feedback types. The free-text type “praise” was less frequently used when the relative either did not feel the patient was cared for with respect and dignity by the doctors (23.4% vs. 40.3%) or if the relative was not supported (7.9% vs. 43.0%); “criticism of care,” on the other hand, was less frequently used when the relative felt nurses and doctors cared for the patient with respect and dignity (51.3% vs. 85.0%, resp. 50.7% vs. 80.4%), and when the relative was supported (48.1% vs. 84.1%); “gratitude” was less frequently expressed when either the relative did not feel the patient was cared for with respect and dignity (4.4% vs. 17.1% resp. 4.5 vs 17.2%) or if they did not feel supported (3.2% vs 18.8%) (Supplement 2 & 3).

### The difference the palliative care unit made

In the survey, 89% of respondents reported they were adequately supported; this was more likely if the patient died on a specialist palliative care unit (odds ratio 6.3; 95% CI, 2.3–17.8) [16]. We found no difference between free-texts from relatives of patients who died in a palliative care unit compared to other wards regarding “praise”, “criticism of care”, and “gratitude” (Supplement 2 & 3), but some categories were more often mentioned in palliative care compared to other units. Relatives who experienced a death occurring on a palliative care unit positively emphasized the “competence”, the “attentive attitude” or the “behavior” of the staff as well as the “place of death” or the “ambience”.

“But the way he was cared for in the last seven days of his life, attentive and ‘professional’, and the way how the last, especially the last 24 hours were for him and us, would somehow justify saying it was a beautiful death.” (Germany, Palliative Care Unit)

Relatives who experienced a death on another ward (rather than a palliative unit) more often rated “information” and “behavior” negatively.

“Communication from ICU physicians was difficult. The staff of the day said one thing, the staff of the night spoke another. There was no agreement between them.” (Brazil, ICU)

## Discussion

Half of the participating relatives in this international post-bereavement survey provided free-text comments, which represented three main types to illustrate their experience: narrative, self-revelation, and feedback. The main themes within these were performance of care, the human factor, communication, environment, and the importance of being a companion to the dying person. From the relatives’ perspective, the staffs’ commitment and attention shaped the experience of end-of-life care, reflected by specific expressions of gratitude in situations where they felt well supported. The free-texts helped to identify and interpret elements of accomplishment as well as gaps of care and support experienced by the relatives.

Our approach aimed not only on *what* the relatives reported, but also *how*, through which we found “gratitude” in the free-texts. Recently, with positive psychology, the concept of gratitude has gained more attention in the context of healthcare [13, 14, 29], especially relating to interventions for palliative care patients [30]. Gratitude may support resilience, providing a way of coping with stressful life events [31] such as those experienced by bereaved relatives. Moreover, the “moral reinforce function” of gratitude [32] is a replicable phenomenon [15, 29], encouraging healthcare professionals to continue with their positive behavior in the future.

Our study does not allow for analysis of the relatives’ motivation for providing gratitude about the care. However, our results suggest that it seems worthwhile to support staff behaviors that generate gratitude in relatives, who represent both current patient interests and those of future service users.

In this context, the emotional experience of care was key, replicating findings of studies exploring the effective elements of palliative care [33, 34]. Both the combination of medical expertise and a considerate, compassionate attitude was appreciated by the relatives in our study. Our quantitative data were in favor of care provided in palliative care units regarding the main outcomes of respect and dignity for the dying person and support for the relative. The qualitative data showed the relatives appreciated the “competence,” the “attentive attitude” or the “behavior” of the staff, and the “place of death” or the “ambience” in the palliative care units. In particular, the “behavior” seemed to make a difference. Negative manifestations of behavior were more frequently reported in wards other than palliative care units, and positive manifestations were more frequently reported in palliative care units. Small gestures such as an understanding glance or a comforting touch had a big impact on the relatives, and good manners conveyed respect for the patient and the relatives, which is similar to previous studies [4, 9, 35–37]. Consequently, attention to patient and relatives’ needs may be a matter of attitude rather than time; although having sufficient time to care may enable a compassionate attitude. Our results about the difference a palliative care unit made, compared with other hospital wards, could support this finding. Additional factors, however, would include the role of specialized training and differing care cultures.

The writing of free-texts showed not only potential for improving the experience of hospital end-of-life care (and bereavement), but also seemed to have a supportive function for grieving relatives. Recalling and writing down their memories represents a way of organizing the past, which in turn can help to alleviate death anxiety in the sense of terror management theory [12, 38], linking to narrative medicine [21]. Our analysis revealed not only themes by content but also themes by type of report, keeping with the sociologist Arthur Frank’s types of illness narrative [39]. His chaos narrative type may fit best to most bereaved relatives’ experience of the loss of order and control while being at the bedside of the dying family member and in the bereavement period. Here, the chaos narrative functions formatively, fixing an order of events in an attempt to take off the pressure, “humanize time” [39, 40], and discover experiences for which relatives are grateful.

We found the grateful experiences were often linked to medical staff, reflecting the relatives’ memory of a healing attachment, the difference the “one person” made. The separate analysis of the 104 Norwegian free-texts from the ERANet-LAC study found, first, attentive care promoting dignity of the dying and, second, the need for impeccable organization such as sufficient staffing and competence levels, coordination, and continuity of care [41]. The two themes were interconnected by the presence of competent and compassionately acting staff managing difficult situations. However, by focusing on the content rather than the representation of the free-texts, the dimension of gratitude did not become apparent. Gray et al. analyzed a comparable structured survey and free-texts from bereaved relatives of patients cared for in US Veterans’ Affairs facilities in 2017 [42]. They organized their analysis in three domains, patient needs, family needs, and facility/ organizational characteristics, and found that patients and family needs are unique. They also found “displays of respect and gratitude for the patient’s life” important for the relatives and the desire to accompany the dying relative.

Our free-texts did not elicit why some relatives reported that they were not informed about the imminent death and about normal alterations during the dying phase of the patient. Several causes may be applicable. Either, doctors and nurses may actually not address these themes; or relatives may be prone to selective listening due to the enormous emotional stress or trauma related to bad news impeding the desire of being a companion to the dying.

### Strengths and limitations

To the best of the authors’ knowledge, this is the first qualitative study on the quality of hospital care for dying cancer patients to be conducted in seven countries across the globe. The free-texts helped us discover what was described as the gap between the biomedical constitution of the disease and the illness experience [43]. We gained rich insight into details of the care experiences from the perspective of the bereaved relatives. These insights cover components which could potentially enhance care and also recognize aspects which impacted in a negative way. Further, we identified different types of free-texts which postulate hypotheses about the potential reasoning and motivation for providing feedback.

The current study is not without limitations. First, different methods of administration of the questionnaires were used. The European countries conducted a postal or electronic survey, while the South American countries collected data via interviews. Thus, response bias and social desirability bias are not fully controlled. Second, due to variations in the translation of i-CODE™, the phrasing used to introduce the space for free-text comments differed in the seven versions of the questionnaire. In Germany, Norway and the UK, a paragraph invited the relative to write a free text, while in Poland and the South American countries only a short sentence offered this possibility, which may have had an impact on the number and richness of detail of the free-texts per country. The way of interviewing as well as the free-text prompts may also have had an impact on the willingness to contribute free-text. These factors constrained a direct comparison between the countries to examine the free-texts for potential cultural differences. However, the themes found in the free-texts were present in all the countries.

### Clinical and research implications

Feedback expressing gratitude seemed to reflect supportive staff behavior contributing to respect and dignity for the dying person. Such behavior was associated with attention and often represented by individual staff members. This requires appropriate training and environmental conditions to enable these attitudes, skills, and behaviors. Engaging with (literary) patient stories could be a useful educational approach to promote patient and family-centered care.

Our study shows that additional qualitative analysis is beneficial even with validated item-based questionnaires, providing a fuller picture of experiences. This type of analysis elicits details behind the item content and unfolds additional dimensions that are important to the respondents. In this context, it seems worthwhile to develop culturally adapted triggers to encourage free-text contributions to give voice to the diverse community of relatives.

Writing free-texts may have a function for relatives in the sense of “terror management” of bereavement, e.g., by discovering elements of care that generate gratitude. Whether relatives’ gratitude may be a surrogate for empathic care appropriate to complement the outcome measurement with i-CODE needs further investigation.

## Conclusion

Our analysis of the survey free-text comments, evaluating quality of care, enabled a better understanding about the findings from the quantitative results. Bereaved relatives highlighted the importance of the perceived support from human interaction between staff and the dying patient and themselves; and that more than professional competence alone, personal, meaningful interactions have profound importance. (Box).

**Table:** 

What bereaved relatives wanted to tell healthcare professionals caring for dying cancer patients in hospitals
• We appreciate your support • We appreciate if we can rely on you acting professionally to preserve the dignity of our dying relative, to relieve pain and other symptoms, to know well your patient, continuously, everybody at any time • We appreciate when you also look after us • We appreciate to be informed about what is going on and what will happen • We appreciate your attention conveyed by small gestures and short actions that make us feel you are present for our dying relative and for us. Stay polite even under pressure, give us a friendly glance, a few compassionate words, offer something to drink, eat, sit down, the opportunity to stay overnight, to be able to accompany our relative in a good way • You are important; every single person at any time has an impact on our experience of accompanying a seriously ill and dying loved one

## Supplementary Information

Below is the link to the electronic supplementary material.

Supplementary file1 (DOCX 31.3 KB)

## Data Availability

Data are archived at the Norwegian Centre for Research Data and can be made available upon reasonable request.

## References

[1] Cohen J, Pivodic L, Miccinesi G, Onwuteaka-Philipsen BD, Naylor WA, Wilson DM, Loucka M, Csikos A, Pardon K, Van den Block L, Ruiz-Ramos M, Cardenas-Turanzas M, Rhee Y, Aubry R, Hunt K, Teno J, Houttekier D, Deliens L (2015). International study of the place of death of people with cancer: a population-level comparison of 14 countries across 4 continents using death certificate data. Br J Cancer.

[2] Cross SH, Warraich HJ (2019). Changes in the place of death in the United States. N Engl J Med.

[3] Pollock K (2015). Is home always the best and preferred place of death?. Br Med J.

[4] Bussmann S, Muders P, Zahrt-Omar CA, Escobar PL, Claus M, Schildmann J, Weber M (2015). Improving end-of-life care in hospitals: a qualitative analysis of bereaved families’ experiences and suggestions. Am J Hosp Palliat Care.

[5] Kupeli N, Candy B, Tamura-Rose G, Schofield G, Webber N, Hicks SE, Floyd T, Vivat B, Sampson EL, Stone P, Aspden T (2019). Tools measuring quality of death, dying, and care, completed after death: systematic review of psychometric properties. Patient.

[6] Brosch T, Scherer KR, Grandjean D, Sander D (2013). The impact of emotion on perception, attention, memory, and decision-making. Swiss Med Wkly.

[7] Phelps EA (2004). Human emotion and memory: interactions of the amygdala and hippocampal complex. Current Opin Neurobiol.

[8] Talarico JM, Rubin DC (2003). Confidence, not consistency, characterizes flashbulb memories. Psychol Sci.

[9] Donnelly S, Prizeman G, Coimin DO, Korn B, Hynes G (2018). Voices that matter: end-of-life care in two acute hospitals from the perspective of bereaved relatives. BMC Palliat Care.

[10] Ó Coimín D, Prizeman G, Korn B, Donnelly S, Hynes G (2019). Dying in acute hospitals: voices of bereaved relatives. BMC Palliat Care.

[11] Greenberg J, Pyszczynski T, Solomon S, Baumeister RF (1986). The causes and consequences of a need for self-esteem: a terror management theory. Public Self and Private Self.

[12] Mikulincer M, Routledge C, Vess M (2019). Chapter 10 - an attachment perspective on managing death concerns. Handbook of Terror Management Theory.

[13] Centeno C, Arantzamendi M, Rodriguez B, Tavares M (2010). Letters from relatives: a source of information providing rich insight into the experience of the family in palliative care. J Palliat Care.

[14] Herbland A, Goldberg MK, Garric N, Lesieur O (2017). Thank you letters from patients in an intensive care unit: from the expression of gratitude to an applied ethic of care. Intens Crit Care Nurs.

[15] Aparicio M, Centeno C, Robinson C, Arantzamendi M (2019). Gratitude between patients and their families and health professionals: a scoping review. J Nurs Manag.

[16] Haugen DF, Hufthammer KO, Gerlach C, Sigurdardottir K, Hansen MIT, Ting G, Tripodoro VA, Goldraij G, Yanneo EG, Leppert W, Wolszczak K, Zambon L, Passarini JN, Saad IAB, Weber M, Ellershaw J, Mayland CR, the E-LACCPG (2021). Good quality care for cancer patients dying in hospitals, but information needs unmet: bereaved relatives’ survey within seven countries. Oncologist.

[17] Mayland CR, Gerlach C, Sigurdardottir K, Hansen MIT, Leppert W, Stachowiak A, Krajewska M, Garcia-Yanneo E, Tripodoro VA, Goldraij G, Weber M, Zambon L, Passarini JN, Saad IB, Ellershaw J, Haugen DF (2019). Assessing quality of care for the dying from the bereaved relatives’ perspective: using pre-testing survey methods across seven countries to develop an international outcome measure. Palliat Med.

[18] Mayland CR, Keetharuth AD, Mukuria C, Haugen DF (2022). Validation of the Care of the Dying Evaluation (i-CODE) within an international study exploring bereaved relatives’ perceptions about quality of care in the last days of life. J Pain Sympt Manag.

[19] Ormston R, Spencer, L.; Barnard M.; Snape, D. (2014) 1. Foundations of qualitative research. In: Ritchie JL, J.; McNaughton Nicholls, C.; Ormston, R. (ed) Qualitative research Practice. SAGE, Thousand Oaks, California, 1–25.

[20] Rehfus WD (2011) UTB Philosophie Wörterbuch. In: UTB Philosophie Wörterbuch. UTB and Vandenhoeck & Ruprecht, Goettingen.

[21] Charon R (2005). Narrative medicine: attention, representation, affiliation. Narrative.

[22] Creswell JW, Klassen AC, Plano Clark VL, Clegg Smith K (2011) Best practices for mixed methods research in the health sciences. In: Best Practices for Mixed Methods Research in the Health Sciences. Office of Behavioral and Social Sciences Research (OBSSR) of the National Institutes of Health (NIH). https://obssr.od.nih.gov/training/online-training-resources/mixed-methods-research/. Accessed 01 October 2016

[23] Vogt A, Stiel S, Heckel M, Goebel S, Mai SS, Seifert A, Gerlach C, Ostgathe C, Weber M (2020). Assessment of the quality of end-of-life care: translation and validation of the German version of the “Care of the Dying Evaluation” (CODE-GER) - a questionnaire for bereaved relatives. Health Qual Life Outcomes.

[24] Gale NK, Heath G, Cameron E, Rashid S, Redwood S (2013). Using the framework method for the analysis of qualitative data in multi-disciplinary health research. BMC Med Res Methodol.

[25] Spencer LR J, O'Connor W, Morrell G, Ormston R (2014) 11. Analysis in practice. In: Ritchie JL, J.; McNaughton Nicholls, C.; Ormston, R. (ed) Qualitative research practice. SAGE, Thousand Oaks, California, 297–345.

[26] Spencer LRJ, Ormston R, O'Connor W, Barnard M (2014) 10. analysis: principles and processes. In: Ritchie JL, J.; McNaughton Nicholls, C.; Ormston, R. (ed) Qualitative research practice. SAGE, Thousand Oaks, California, pp. 269–293.

[27] Farquhar M, Preston N, Evans CJ, Grande G, Short V, Benalia H, Higginson IJ, Todd C, Morecare (2013). Mixed methods research in the development and evaluation of complex interventions in palliative and end-of-life care: report on the MORECare consensus exercise. J Palliat Med.

[28] Brennan RL, Prediger DJ (1981). Coefficient kappa: some uses, misuses, and alternatives. Educ Psychol Meas.

[29] Day G, Robert G, Rafferty AM (2020). Gratitude in health care: a meta-narrative review. Qual Health Res.

[30] Althaus B, Borasio GD, Bernard M (2018). Gratitude at the end of life: a promising lead for palliative care. J Palliat Med.

[31] McCullough ME, Emmons RA, Tsang J-A (2002). The grateful disposition: a conceptual and empirical topography. J Pers Soc Psychol.

[32] McCullough ME, Kilpatrick SD, Emmons RA, Larson DB (2001). Is gratitude a moral affect?. Psychol Bull.

[33] Black A, McGlinchey T, Gambles M, Ellershaw J, Mayland CR (2018). The ‘lived experience’ of palliative care patients in one acute hospital setting – a qualitative study. BMC Palliat Care.

[34] Sampson C, Finlay I, Byrne A, Snow V, Nelson A (2014). The practice of palliative care from the perspective of patients and carers. BMJ Support Palliat Care.

[35] Mayland CR, Mulholland H, Gambles M, Ellershaw J, Stewart K (2017). How well do we currently care for our dying patients in acute hospitals: the views of the bereaved relatives?. BMJ Support Palliat Care.

[36] Rogers A, Karlsen S, Addington-Hall J (2000). ‘All the services were excellent. It is when the human element comes in that things go wrong’: dissatisfaction with hospital care in the last year of life. J Adv Nurs.

[37] Virdun C, Luckett T, Davidson PM, Phillips J (2015). Dying in the hospital setting: a systematic review of quantitative studies identifying the elements of end-of-life care that patients and their families rank as being most important. Palliat Med.

[38] Kosloff S, Anderson G, Nottbohm A, Hoshiko B, Routledge C, Vess M (2019). Chapter 2 - proximal and distal terror management defenses: a systematic review and analysis. Handbook of terror management theory.

[39] Frank AW (2004). Asking the right question about pain: narrative and phronesis. Lit Med.

[40] Kermode F (1967). The sense of an ending: studies in the theory of fiction.

[41] Hansen MIT, Haugen DF, Sigurdardottir KR, Kvikstad A, Mayland CR, Schaufel MA, group ER-LCp (2020). Factors affecting quality of end-of-life hospital care - a qualitative analysis of free text comments from the i-CODE survey in Norway. BMC Palliat Care.

[42] Gray C, Yefimova M, McCaa M, Goebel JR, Shreve S, Lorenz KA, Giannitrapani K (2020). Developing unique insights from narrative responses to bereaved family surveys. J Pain Symptom Manage.

[43] Kleinman A (1988) The illness narratives suffering, healing, and the human condition. Basic Books, New York, 3, 5.

