# Supplementary material for: What do bereaved relatives of cancer patients dying in hospital want to tell us? Analysis of free-text comments from the International Care of the Dying Evaluation (i-CODE) survey: a mixed methods approach
Source: Support Care Cancer. 2022 Dec 23;31(1):81. doi: 10.1007/s00520-022-07490-9 (PMC9788999; doi:10.1007/s00520-022-07490-9)
Supplement: Supplementary file 1 — Supplementary file1 (DOCX 31.3 KB) [file 520_2022_7490_MOESM1_ESM.docx]

**Supplement 1: Varying prompts introducing the free-text space.** English translations of the text introducing the free-text space in the 7 countries

| **country** | **Original version of the text introducing the free-text field of the i-CODE questionnaire** | **English translation of the text introducing the free-text field of the i-CODE questionnaire** |
| --- | --- | --- |
| Argentina | Si lo desea, siéntase en libertad de hacer comentarios acerca de cualquier aspecto de la atención y el apoyo recibidos en general: | Please feel free to comment on any aspect of the overall care and support received. |
| Brasil | Sinta-se à vontade para fazer quaisquer comentários sobre sua impressão geral dos cuidados e apoio recebidos: | Feel free to provide any comments about your overall impression of the care and support received: |
| Germany | Auch wenn der Fokus dieses Fragebogens bisher sehr stark auf ihren/seinen letzten Lebenstagen gelegen hat, ist uns bewusst, dass es weitere Aspekte der Betreuung und Unterstützung vor diesem Zeitraum geben kann, über die Sie uns eine Rückmeldung geben möchten. Hier haben Sie die Möglichkeit, sich zu weiteren Aspekten der Gesamtbetreuung und Unterstützung zu äußern, die stattgefunden hat. | Although the focus of this questionnaire so far has been very much on his/her last days of life, we are aware that there may be other aspects of care and support prior to this period that you would like to provide feedback on. Here is your opportunity to comment on other aspects of the overall care and support that took place. |
| Norway | Selv om fokuset i dette spørreskjemaet har vært svært mye på hans/ hennes siste levedager, er vi klar over at det kan være andre forhold ved behandling, pleie eller støtte før perioden, som du kan vaereønske å gi tilbakemelding om. Hvis du ønsker det, føl deg fri til å kommentere på hvilket som helst forhold ved behandlingen, pleien og sttten dere fikk; både tidligere i forløpet og I de siste to dagene. Legg gjerne ved et ekstra ark, om du ønsker det. | Although the focus of this questionnaire has been very much on his/her  last days of life, we are aware that there may be other aspects of  treatment, care and support prior to this period that you would like to  provide feedback on. If you so wish, feel free to comment on any aspect  related to the treatment, care and support given to you; both earlier in the  trajectory and in the last two days of life. If you wish, you may well  enclose an additional sheet of paper. |
| Poland | Uwagi | comments |
| United Kingdom | Although the focus of this questionnaire has very much been on the last days of life, we appreciate there may be other aspects of care or support prior to this time, which you wish to feedback. Please feel free to comment, if you wish to, on any aspect of the overall care and support received: | Although the focus of this questionnaire has very much been on the last days of life, we appreciate there may be other aspects of care or support prior to this time, which you wish to feedback. Please feel free to comment, if you wish to, on any aspect of the overall care and support received: |
| Uruguay | Si lo desea, siéntase en libertad de hacer comentarios acerca de cualquier aspecto de la atención y el apoyo recibidos en general: | Please feel free to comment on any aspect of the overall care and support received. |

Due to local conditions, the data collection was via postal survey in Germany, Norway, the U.K., and face-to-face interview in Argentina, Brasil, Uruguay, and Poland (with tablet).

**Supplement 2: Absolute numbers and percentage of persons who gave feed backs to gratitude free-texts and sociodemographic data, place of care, and primary questionnaire outcomes.**

|  | **‘gratitude’** | | | **p-value** |
| --- | --- | --- | --- | --- |
|  | **Yes** | **No** | **total** |  |
| **total** | 72 (15.8) | 385 (84.2) | 457 (100%)**^4^** |  |
| **gender (relatives)**  male  female  Missing Data | 24 (17.1 %)  47 (15.2 %)  1 (1.4 %) | 116 (82.9 %)  263 (84.8 %)  6 (1.6 %) | 140 (30.6 %)  310 (67.8 %)  7 (1.5 %) | 0.8621 |
| **age (relatives)**  <60  60–79  ≥80  Missing Data | 44 (17.1 %)  26 (14.8 %)  2 (10.0 %)  0 (0.0 %) | 213 (82.9 %)  150 (85.2 %)  18 (90.0 %)  4 (1.0 %) | 257 (56.3 %)  176 (38.5 %)  20 (4.4 %)  4 (0.9 %) | 0.6289 |
| **relationship**  partner  daughter/son in law  other  Missing Data | 29 (14.4 %)  34 (19.1 %)  9 (12.2 %)  0 (0.0 %) | 173 (85.6 %)  144 (80.9 %)  65 (87.8 %)  3 (0.8 %) | 202 (44.2%)  178 (39.0 %)  74 (16.2 %)  3 (0.7 %) | 0.3795 |
| **palliative care unit**  Yes  No | 22 (18.9 %)  50 (14.2 %) | 84 (79.2 %)  301 (85.8 %) | 106 (23.2 %)  351 (76.8 %) | 0.1069 |
| **respect and dignity - nurses^1^**  Yes  No  Missing Data/I do not know | 71 (17.2 %)  1 (2.5 %)  0 (0.0 %) | 342 (82.8 %)  39 (97.5 %)  4 (1.0 %) | 413 (90.4 %)  40 (8.8 %)  4 (0.9 %) | 0.0354 |
| **respect and dignity - doctors^2^**  Yes  No  Missing Data/I do not know | 66 (17.1 %)  2 (4.4 %)  4 (5.6 %) | 321 (82.9 %)  44 (95.6 %)  20 (5.2 %) | 387 (84.7 %)  46 (10.1 %)  24 (5.3 %) | 0.0814 |
| **support for the relative^3^**  Yes  No  Missing Data/I do not know | 70 (18.8 %)  2 (3.2 %)  0 (0.0 %) | 302 (81.2 %)  61 (96.8 %)  22 (5.7 %) | 372 (81.4 %)  63 (13.8 %)  22 (4.8 %) | 0.0008 |

^1^ Q30: How much of the time was s/he treated with respect and dignity in the last two days of life? (nurses)

^2^ Q30: How much of the time was s/he treated with respect and dignity in the last two days of life? (doctors); ‘Yes’ for ‘always’ and ‘most of the time’; ‘No’ for ‘some of the time’ and ‘never’

^3^ Q31: Overall, in your opinion, were you adequately supported during his/her last two days of life?

^4^ % in total-columns referring to n = 457, all other % refer to the use of free-text in the palliative care unit, and in correlation with the primary outcome items

**Supplement 3: Absolute numbers and percentage of persons who gave feed-back free-texts containing praise and criticism of care according to hospitalization in palliative care unit and to primary questionnaire outcomes.**

|  | **Use of free-text ‘praise’** | | | **p-value** | **Use of free-text ‘criticism of care’** | | | **p-value** |
| --- | --- | --- | --- | --- | --- | --- | --- | --- |
|  | **yes** | **no** | **total^4^** |  | **yes** | **no** | **total^4^** |  |
| **total** | 172 (37.6 %) | 285 (62.4 %) | 457 (100 %) |  | 249 (54.5%) | 208 (45.5%) | 457 (100%) |  |
| **palliative care unit**  Yes  No | 45 (42.5 %)  127 (32.2 %) | 61 (57.5 %)  224 (63.8 %) | 106 (23.2 %)  351 (76.8 %) | 0.2429 | 57 (53.8 %)  192 (54.7 %) | 49 (46.2 %)  159 (45.3 %) | 106 (23.2 %)  351 (76.8 %) | 0.8666 |
| **respect and dignity - nurses^1^**  Yes  No  Missing Data/I do not know | 162 (39.2 %)  10 (25.0 %)  0 (0.0 %) | 251 (60.8 %)  30 (75.0 %)  4 (1.4 %) | 413 (90.4 %)  40 (8.8 %)  4 (0.9 %) | 0.0614 | 212 (51.3 %)  34 (85.0 %)  3 (1.2 %) | 201 (48.7 %)  6 (15.0 %)  1 (0.5 %) | 413 (90.4 %)  40 (8.8 %)  4 (0.9 %) | 0.0002 |
| **respect and dignity - doctors^2^**  Yes  No  Missing Data/I do not know | 156 (40.3 %)  11 (23.9 %)  5 (2.9 %) | 231 (59.7 %)  35 (7.6 %)  19 (6.7 %) | 387 (84.7 %)  46 (10.1 %)  24 (5.3 %) | 0.0207 | 196 (50.7 %)  37 (80.4 %)  16 (6.4 %) | 191 (49.4 %) 9 (19.6 %)  8 (3.9 %) | 387 (84.7 %)  46 (10.1 %)  24 (5.3 %) | 0.0003 |
| **support for the relative^3^**  Yes  No  Missing Data/I do not know | 160 (43.0 %)  5 (7.9 %)  7 (4.1 %) | 212 (74.4 %)  58 (20.4 %)  15 (5.3 %) | 372 (81.4 %)  63 (13.8 %)  22 (4.8 %) | <0.0001 | 179 (48.1 %)  53 (84.1 %)  17 (6.8 %) | 193 (51.9 %)  10 (45.4 %)  5 (2.4 %) | 372 (81.4 %)  63 (13.8 %)  22 (4.8 %) | <0.0001 |

^1^ Q30: How much of the time was s/he treated with respect and dignity in the last two days of life? (nurses)

^2^ Q30: How much of the time was s/he treated with respect and dignity in the last two days of life? (doctors); ‘Yes’ for ‘always’ and ‘most of the time’; ‘No’ for ‘some of the time’ and ‘never’

^3^ Q31: Overall, in your opinion, were you adequately supported during his/her last two days of life?

^4^ % in total-columns referring to n = 457, all other % refer to the use of free-text in the palliative care unit, and in correlation with the primary outcome items

**Supplement 4 to the typology hierarchy (Table 2).** Hierarchical portrayal of the typological categories and subcategories including the definitions of the categories supplemented by anchor quotations to illustrate the meaning of the category.

| **Category** | **Subcategory** | **Definition** | **Anchor quotation** |
| --- | --- | --- | --- |
| Feedback | Praise | The text is used to highlight positive experiences made in the hospital or regarding the dying phase. | *“The care which the patients and families received from the entire medical staff like nurses was very attentive and professional.”* Argentina, regular ward |
|  | Suggestions/demands for improvement* | Ideas by the relative how the hospital/staff could improve its work. This can be a direct suggestion or provided in an indirect way i.e. complaint about issues that enable conclusions to be made about how to improve patient care and relative support. | *“A support group for the families of those who are dying would be helpful. Improve general housing conditions”* Poland, Palliative Care Unit |
|  | Burden due to negative experience* | The relative feels an emotional burden caused by the experience of poor care or inappropriate circumstances (e. g. the organizational structures of the hospital) during the hospital stay. The poor care or inappropriate circumstances are distressing explicitly attributed experience of the relative. | *“However, it is a great burden to me that I was not informed in time of the death of my [wife] during her last stay in the [NAME hospital] so that I could not support her during the last hours of her life.”* Germany, Haematology Ward |
|  | Gratitude | The relative uses the opportunity to explicitly say ‘thank you’ or to express their gratitude for the care, to the team or to a certain person. | *“I thank you all very much. I was going to write a letter of thanks, but since I came here, I ask you to give my thanks to all the doctors and nurses who have treated us with great respect.”* Brazil, Medical Ward |
|  | Complaint* | The text is used to report concrete negative experiences made in the hospital or regarding the dying phase, but the content of the free text does not allow for further interpretation regarding emotional processing, or improvement ideas. | *“Given that my husband was diagnosed with probable liver cancer on (date) and was kept in hospital until (7 days later), the only pain relief he was given on his discharge was  paracetamol, which in my opinion was totally inadequate. On (5 days later) he was in that much pain that I had to call 111 who in turn sent an ambulance, and he was given morphine on the way to the hospital. I feel that on his discharge on (5 days later) this should have been given as who knows how much pain he put up with before he asked for me to call 111, and for him to  suggest that I knew it must be bad”* UK, Palliative Care Unit |
|  | Feedback to questionnaire | The relative suggests how the questionnaire could be improved. | *“The answer options in the survey are differently constructed and partly poorly balanced (see*  *question 20; 3 options for «good» and 1 for «bad»)”* Norway, Medical Ward |
| Self-revelation | Social context | The relative reveals personal information about himself/herself or the patient. | *“My husband died in the emergency room, because the circumstances to say goodbye at home were not favorable: children in our care.”* Uruguay, Emergency Room |
|  | Coping with bereavement | The relative explains how s/he is dealing with the loss or how the time since the death has been for him/her. | *“The time afterwards has been and still is EXTREMELY hard for me, but probably it has to be that way?”* Norway, Palliative Care Unit |
|  | Impact on health/ psyche | The burden caused by being an informal caregiver has a negative impact on the relative's health or emotional well-being. This impact can be described as (mental/ physical) exhaustion or sadness. | *“He did have a very long and sad disease-CV, so the years have not been good, neither for him nor for me as next-of-kin. There were cases of mistreatment, and poor understanding for his health condition. We were frustrated and sorry more than once. So this has also made its marks on my own health condition.”* Norway, Oncology Ward |
|  | Rage attributed to negative experience | The relative expresses strong feelings such as rage attributed to negative experiences about care, communication or hospital procedures. | „My partner's cervical biopsy was misjudged, so she got cancer. The cancer treatment at the hospital [name] did not meet our expectations, as we had to buy the cancer drugs ourselves several times at our own expense (recommended by the hospital's oncologist). This happened despite the fact that the hospital was responsible for causing the cancer and ultimately killing my partner and the mother of two children.*“* Norway, Palliative Care Unit |
|  | ‘Kitchen table’ wisdom | This category collects wisdom from real life experiences which the relative shares in the free texts. | *“We would like to emphasize the importance of palliative care. Knowing that the death of someone you love is near is suffered and no one is prepared.”* Brazil, Medical Ward |
| Narrative | Chronicle | Chronological description or report of the patient's history including hospital stay, therapies and decline. | *“Until Christmas 2017 my husband seemed to be stable and healthy. On 26/12 he was admitted to the hospital by the emergency doctor. On 28/12 we got the devastating diagnosis: Acute leukemia. On 29/12 he was admitted to [Hospital]. On New year’s eve his condition was critical. He was stabilized and the doctors recommended chemo therapy. He was cared for very well in the hematology ward. Doctors and caregivers were very friendly, they gave good advice and we were well supported. The therapy took effect. Due to germs his condition went south. …”* Germany, Haematology Ward |
|  | Witnessing the imminently dying | Report of the imminently dying phase. | *“I told my son that I would not leave him alone, and that I would stay with him. Then he became calm. A short time before he did his last breath he opened wide his eyes, looked at me, looked thorough me and to the roof. I told him, that now the time has come, when our dear departed came for him. He died in my arms.”* Germany, Oncology Ward |

Title: What do bereaved relatives want to tell us? Analysis of free-text comments from Care of the Dying Evaluation International Survey (i-CODE) – a mixed methods approach

Journal: Support Care Cancer

Autors: Christina Gerlach*, Miriam Baus, Emilio Gianicolo, Oliver Bayer, Dagny Faksvåg Haugen, Martin Weber, Catriona R Mayland

*Corresponding author: christina.gerlach@med.uni-heidelberg.de

1. University Medical Center of Johannes Gutenberg University, Interdisciplinary Palliative Care Unit, Department of Medicine, Mainz, Germany

2. Heidelberg University Hospital, Department of Palliative Care, Heidelberg, Germany
